# *Boehmiella wilsoni* (Nematoda, Heligmosomoidea, Boehmiellidae fam. nov.), found in Amazonian rodents

**DOI:** 10.1016/j.ijppaw.2020.08.003

**Published:** 2020-08-27

**Authors:** B.E. Andrade-Silva, R.V. Vilela, E.J. Lopes-Torres, S.F. Costa-Neto, A. Maldonado

**Affiliations:** a Programa de Pós-graduação em Biologia Parasitária, Instituto Oswaldo Cruz, Fundação Oswaldo Cruz, Brazil; b Laboratório de Biologia e Parasitologia de Mamíferos Silvestres Reservatórios, Instituto Oswaldo Cruz, Fundação Oswaldo Cruz, Brazil; c Laboratório de Helmintologia Romero Lascasas Porto, Departamento de Microbiologia, Imunologia EParasitologia, Faculdade de Ciências Médicas, Centro Biomédico, Universidade Do Estado Do Rio de Janeiro – UERJ, Brazil; d Campus Fiocruz Mata Atlântica, Fundação Oswaldo Cruz – FIOCRUZ, Rio de Janeiro, RJ, Brazil

**Keywords:** Scanning electron microscopy, Molecular phylogenetics, 18S rRNA, 28, Echimyidae

## Abstract

The genus *Boehmiella* was initially described as a member of the family Trichostrongylidae. Subsequently, it was assigned to the subfamily Haemonchinae in the family Haemonchidae. We analyzed parasites of spiny tree-rats, *Mesomys hispidus*, collected in the Amazon rainforest, which were identified as *B. wilsoni* based on integrative taxonomy. Using morphology, morphometry, and scanning electron microscopy (SEM), we added new data to the original description of the species. We also inferred phylogenetic hypotheses for its relationships within the Trichostrongylina, based on partial nuclear 18S and 28S rRNA genes, through Maximum Likelihood and Bayesian analyses. In conclusion, *B. wilsoni* does not belong to the family Haemonchidae, nor is it closely related to any other trichostrongylin family, and therefore, we propose the establishment of a new family, Boehmiellidae fam. nov., to which the genus *Boehmiella* is allocated.

## Highlights

• Helminth found in arboreal rodent of the Amazon region, with description of new host, new geographical distribution, new taxonomic data, and proposal of a new family to allocate the genus according to molecular and morphological analyses.

## Graphical abstract

**Figure:**
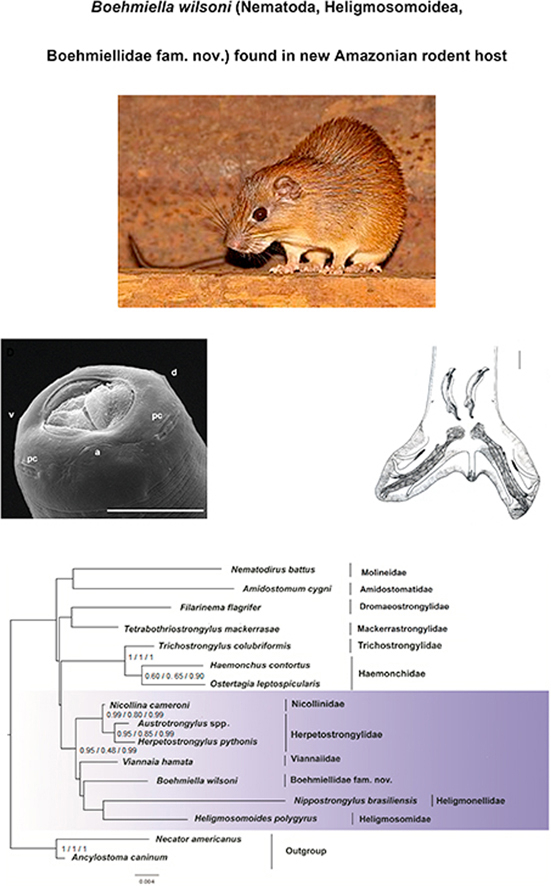

## 1 Introduction

The genus *Boehmiella* Gebauer, 1932 was described initially as a member of the family Trichostrongylidae Leiper, 1912. The type species, *Boehmiella perichitinea* Gebauer (1932), was first reported from a German zoo as a parasite of the rodent *Myocastor coypus* (G. I. Molina, 1782), and was later found in this same host in both Brazil and Argentina (Lent and Freitas, 1934; Martinez et al., 2004). A second species, *Boehmiella wilsoni* Lucker (1943), was described parasitizing the grey squirrel, *Sciurus carolinensis* Gmelin, 1788, in the United States. More recently, *B. wilsoni* has been found in *Sciurus deppei* Peters, 1863 in Mexico (Falcon-Ordáz and García-Prieto, 2004); in *Cuniculus paca* (Linnaeus, 1766) in Peru (Baquedano, 2014); and in *Dasyprocta variegata* Tschudi, 1845 in Bolivia (Mollericona et al., 2016).

Gebauer (1932) diagnosed the genus *Boehmiella* based on the developed neodont that emerges from the anterior part of the esophagus, followed by two pairs of denticles located in the lumen of the buccal cavity, four cephalic papillae, and two derids. The species lacks a buccal capsule and cephalic vesicle, has highly sclerotised lateral rays of the copulatory bursa, a gubernaculum, and a vulva, located posteriorly in the body.

However, Travassos (1937) disagreed on the allocation of the genus *Boehmiella* to the family Trichostrongylidae and concluded that a more detailed study might allocate the genus to a major new group. Subsequently, Yamaguti (1961) proposed the establishment of a new subfamily, the Boehmiellinae, to accommodate the genus *Boehmiella*, based on the sclerotization of the lateral rays of the copulatory bursa. However, Durette-Desset et al. (1999) did not consider this single morphological feature sufficient to support the subfamily Boehmiellinae and established the current classification, in which *Boehmiella* is included in the subfamily Haemonchinae Skrjabin and Schulz, 1952. These authors also proposed hypotheses related to the evolutionary history of the Trichostrongyloidea, based on a cladistic analysis, and concluded that, although *Boehmiella* was first described in Germany, the genus would have emerged in the Nearctic region, during the upper Miocene, coinciding with the migration of squirrels (Sciuridae) to North America, before dispersing throughout the Holarctic region and only recently *Boehmiella* would have begun parasitizing *M. coypus*, after this caviomorph was introduced to the Holarctic region.

In this study, we report *B. wilsoni* from the Brazilian Amazon for the first time, providing an expansion of the geographical distribution of this species, as also a new host: the caviomorph echimyid rodent Ferreira's spiny tree-rat, *Mesomys hispidus* (Desmarest, 1817). Our study includes morphological and morphometric data and a new ultrastructural description using the scanning electron microscopy (SEM). Based on our molecular phylogenies, we conclude that the species *B. wilsoni* does not group with the species of the genera *Haemonchus* Cobb, 1898 and *Ostertagia* Ransom, 1907, and therefore, *Boehmiella* should not be assigned to the family Haemonchidae (Skrjabin and Schulz, 1937). In the light of these findings, we propose a new family, the Boehmiellidae fam. nov., to accommodate the genus *Boehmiella,* based on an integrated taxonomic approach, using morphological, ultrastructural, and molecular tools.

## 2 Material and methods

### 2.1 Host collection

Three specimens of the caviomorph echimyid rodent, *Mesomys hispidus* (Desmarest, 1817), were captured in the municipality of Senador Guiomard, in the state of Acre, Brazil (10°09′39.0″S; 67°44′17.6″W), in December 2016, using Sherman trapsmodel XLK (H.B. Sherman Traps, Tallahassee, Florida); baited with a mixture of peanut butter, banana, oats, and bacon. The collection of animal specimens was authorized by the Chico Mendes Institute for Biodiversity Conservation - ICMBio (*Instituto Chico Mendes de Conservação da Biodiversidade*) of the Brazilian federal government, through permit N° 13,373. Capture and handling procedures followed the guidelines of the Ethics Committee for the experimental Use of Animals (CEUA) of the Oswaldo Cruz Institute - IOC (*Instituto Oswaldo Cruz*), authorization number L-39/14. They were anesthetized and euthanized for the collection of helminths and other biological samples. All biological sampling procedures were conducted using appropriate biosafety practices (Lemos and D'Andrea, 2014). *M. hispidus* voucher specimens were deposited in the scientific collection of the National Museum of Brazil, Federal University of Rio de Janeiro (*MN/UFRJ*).

### 2.2 Studies on the helminth parasites

The parasitic worms recovered from the mammal specimens were washed in a 0.85% saline (NaCl) solution and stored in 70% ethanol. For light microscopy, the nematodes were cleared in lactophenol and drawings were produced with the aid of a camera lucida attached to a Zeiss Scope Z1 light microscope (Zeiss, Göttingen, Germany). The observed structures were measured from digital images captured by a Zeiss Axio Cam HRC (Zeiss, Germany), using the Carl Zeiss AxioVision Rel. 4.7 accessory software. All measurements are shown in millimeters.

For scanning electron microscopy (SEM), four fixed specimens (two males and two females) were processed according to a protocol modified from Souza et al. (2017). The helminths were dehydrated in a 70%–absolute ethanol gradient. First, the samples were dehydrated in 70% ethanol for 48 h and then 80%, 90%, and absolute ethanol for 20 min, at each step. Finally, the samples were dried in aliquid CO_2_ critical point drying machine, mounted on metal stubs and coated with gold (20 nm). Samples were analyzed using a Thermo-Fisher Quanta 250 scanning electron microscope in the Microscopy Division of the National Institute of Science and Technology for Structural Biology and Bio-imagery - CENABIO/UFRJ.

The helminth nomenclature followed Gebauer (1932), Lucker (1943), Falcon-Ordáz and García-Prieto (2004) and Mollericona et al. (2016). Specimens were deposited in the Helminthological Collection of the Oswaldo Cruz Institute – CHIOC (*Coleção Helmintológica do Instituto Oswaldo Cruz*) under catalog number CHIOC: 38568.

### 2.3 Molecular phylogenetic analyses

Genomic DNA was isolated from one specimen using the QIAamp DNA Mini Kit (QIAGEN, Hilden, Germany), following the manufacturer's protocol. DNA was amplified by polymerase chain reaction (PCR) using a pair of primers for the small subunit ribosomal RNA (18S rRNA) gene (Gomes et al., 2015) and seven primer pairs for the large subunit ribosomal RNA (28S rRNA) gene (Chilton et al., 2003). Each PCR contained 12.5 μL of PCR Master Mix (Promega Corporation, Madison, USA), 8.5 μL of DNA-free water, 0.5 μL of each forward and reverse primers, and 3 μL of the DNA sample in a total reaction volume of 25 μL. PCR cycling parameters followed Gomes et al. (2015), for the 18S rRNA gene and Chilton et al. (2003), for the 28S rRNA gene. The resulting amplicons were electrophoresed in 1.5% agarose gel using Gel Red™ nucleic acid gel stain (Biotium, Hayward, California, USA), and visualized in a UV transilluminator. Successfully amplified amplicons were purified using the illustra GFX PCR DNA and Gel Band Purification Kit (GE Healthcare, Little Chalfont, UK), according to the manufacturer's protocol. Amplicons were cycle-sequenced using the Big Dye Terminator v3.1 Cycle Sequencing kit (Applied Biosystems, USA). Both strands were sequenced to ensure accuracy. Samples were sequenced in an ABI3730 DNA Analyzer. All sample processing and sequencing was conducted at the DNA Sequencing Platform of the Oswaldo Cruz Institute - PDTIS/Fiocruz (*Plataforma de Sequenciamento de DNA do Instituto Oswaldo Cruz*). Sequence fragments were assembled into contigs and edited for ambiguities using Geneious 9.1.8 (Kearse et al., 2012) to provide consensus sequences.

In addition to the consensus sequences of both the 18S and the 28S ribosomal RNA (rRNA) genes of *B. wilsoni*, we also obtained the 28S rRNA gene sequence of a specimen of *Viannaia hamata* Travassos, 1914, recovered from a marsupial *Didelphis aurita* Weid-Neuweid, 1826, from Porto Alegre, in the state of Rio Grande do Sul. We aligned our 18S rRNA gene sequence of *B. wilsoni* with 24 sequences of other nematode species belonging to the suborder Trichostrongylina (*sensu* Durette-Desset and Chabaud, 1993) retrieved from GenBank (Table 1). In the case of our 28S rRNA gene sequences, we aligned our sequences of *B. wilsoni* and *V. hamata* with 33 sequences of Trichostrongylina retrieved from (Table 1). As outgroups for both datasets (18S and 28S rRNA), we used two sequences of nematode species belonging to the suborder Ancylostomatina (*Ancylostoma caninum* (Ercolani, 1859) and *Necator americanus* Stiles, 1092).

**Table 1:** List of species and the GenBank accession numbers of the sequences included in the present study.

Family	Subfamily	Species	28S rRNA	18S rRNA
Amidostomatidae	Amidostomatinae	*Amidostomum cygni*	AM039745	AJ920353
Ancylostomatidae	Ancylostomatinae	*Ancylostoma caninum*	AM039739	AJ920347
	Bunostominae	*Necator americanus*	AM039740	AJ920348
Cooperiidae	Cooperiinae	*Cooperia curticei*	LN715235	–
	Libyostrongylinae	*Libyostrongylus douglassi*	LN715233	–
Dromaeostrongylidae	Dromaeostrongylinae	*Dromaeostrongylus bicuspis*	LN715218	–
	Filarinematinae	*Filarinema flagrifer*	AM039746	AJ920354
		*Peramelistrongylus skedastos*	LN715222	–
Haemonchidae	Haemonchinae	*Haemonchus contortus*	AM039742	EU086374
	Ostertagiinae	*Camelostrongylus mentulatus*	LN715234	–
		*Graphidium strigosum*	LN715219	–
		*Hyostrongylus rubidus*	LN715237	–
		*Ostertagia leptospicularis*	AM039744	AJ920351
		*Ostertagia ostertagi*	–	AF036598
		*Teladorsagia circumcincta*	LN715236	–
Heligmonellidae	Nippostrongylinae	*Carolinensis perezponcedeleoni*	–	JX877672
		*Hassalstrongylus* sp.	–	JX877679
		*Nippostrongylus brasiliensis*	LN715229	AJ920356
		*Nippostrongylus magnus*	AM039748	–
		*Odilia bainae*	LN846131	–
Heligmosomidae	Heligmosominae	*Heligmosomoides polygyrus*	AM039747	AJ920355
Herpetostrongylidae	Globocephaloidinae	*Amphicephaloides thylogale*	LN715232	–
		*Globocephaloides macropodis*	LN715231	–
	Herpetostrongylinae	*Austrostrongylus chandleri*	LN715224	–
		*Austrostrongylus victoriensis*	–	JX877684
		*Beveridgiella iota*	LN715228	–
		*Herpetostrongylus pythonis*	AM039750	AJ920358
		*Paraustrostrongylus bettongia*	LN715226	–
		*Patricialina hickmani*	LN715227	–
		*Sutarostrongylus johnsoni*	LN715225	–
		*Woolleya monodelphis*	LN846132	–
Mackerrastrongylidae	Mackerrastrongylinae	*Mackerrastrongylus isoodon*	LN715221	–
		*Tetrabothriostrongylus mackerrasae*	AM039751	AJ920359
	Tasmanematinae	*Tachynema baylisi*	LN715223	–
Molineidae	Molineinae	*Oswaldocruzia* sp.	–	JX877669
	Nematodirinae	*Nematodirella cameli*	–	JX305977
		*Nematodirus battus*	AM039752	AJ920360
		*Nematodirus helvetianus*	LN715238	–
	Ollulaninae	*Ollulanus tricuspis*	LN715220	–
Nicollinidae		*Nicollina cameroni*	AM039749	AJ920357
Ornithostrongylidae	Ornithostrongylinae	*Vexillata convoluta*	–	JX877672
Trichostrongylidae	Trichostrongylinae	*Trichostrongylus colubriformis*	AM039743	AJ920350
Viannaiidae	Viannaiinae	*Travassostrongylus callis*	–	JX877677
		*Travassostrongylus orlofi*	–	JX877671
		*Viannaia didelphis*	–	JX877676
		*Viannaia minispicula*	–	JX877682
		*Viannaia hamata*	–	JX877680

We aligned sequences of each dataset using the ClustalW multiple sequence alignment program (Thompson et al., 1994). We trimmed poorly aligned regions using the Mesquite software package, version 3.51 (Maddison and Maddison, 2018). Uncorrected pairwise *p*-distances were calculated for each matrix (18S and 28S) using PAUP*, version 4.0a164 (Swofford, 2002). Nucleotide substitution saturation in each matrix was assessed using the test by Xia et al. (Xia et al., 2003; Xia and Lemey, 2009) executed in DAMBE, version 6.4.79 (Xia and Xie, 2001). We also used Mesquite to build a concatenated matrix of the 18S and 28S rRNA genes, only utilizing samples for which sequences were available for both genes, a total of 16 sequences (Table 1).

For each matrix (18S, 28S, and concatenated), we conducted Maximum Likelihood (ML) phylogenetic reconstructions using PhyML 3.0 (Guindon et al., 2010). Substitution models were calculated using Smart Model Selection (SMS) in PhyML (Lefort et al., 2017), under the Akaike information criterion (AIC). The nodes robustness was assessed by Approximate Likelihood-Ratio Test for Branches (aLRT) (Anisimova and Gascuel, 2006) and by nonparametric bootstrap percentages (ML-BP), with 1,000 pseudoreplications, both implemented in PhyML 3.0.

We conducted Bayesian phylogenetic inference (BI) phylogenetic reconstructions using MrBayes 3.2.6 (Ronquist et al., 2012), on the XSEDE platform through the CIPRES Science Gateway (Miller et al., 2010), for each matrix. Substitution models were calculated and implemented separately for each partition (18S and 28S) using the automated model selection in PAUP*, version 4.0a164 (Swofford, 2002), under the Bayesian information criterion (BIC). We sampled MCMC for 10,000,000 generations, with four simultaneous chains, in two runs, at every 100 generations, after discarding an initial burn-in of 25%. The nodes robustness was assessed using Bayesian posterior probabilities (BPP) calculated from the sampled trees. To assess the BI sampling adequacy, we used Tracer v1.6 (Rambaut et al., 2014) to calculate the Effective Sample Sizes (ESSs) of each parameter. We considered values of over 100 effectively independent samples as adequate.

## 3 Results

### 3.1 Morphological analyses using light and scanning electron microscopy

**Fig. 1:**
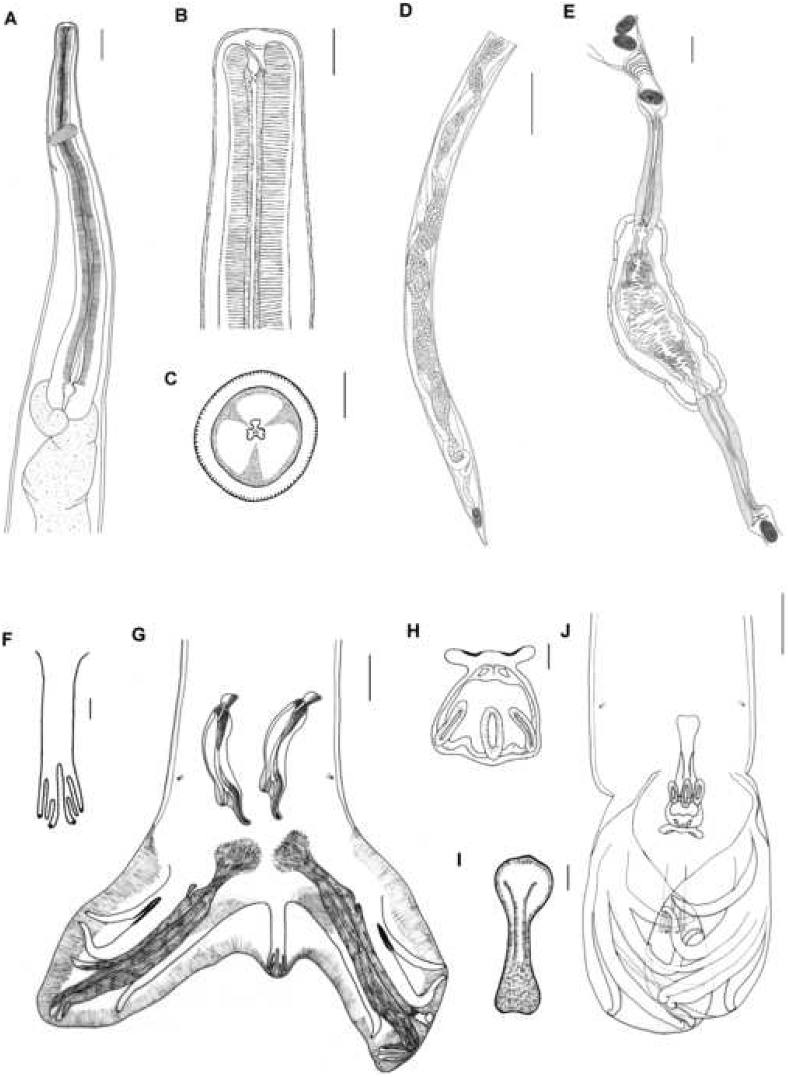
Light microscopy with camera lucida of *Boehmiella wilsoni.* (A) Anterior part of female body. (B) Neodont. (C) Cross-section of the head, with the neodont and denticles in detail. (D) Posterior part of female body. (E) Dissected ovejector. (F) Dorsal rays. (G) Posterior part of male body. (H) Telamon. (I) Gubernaculum. (J) Posterior part of male body, copulatory bursal closed.

The *Boehmiella* specimens analyzed in the present study were identified based on Gibbons and Khalil (1982). Anterior extremity of males and females with small head, lips and cephalic capsule absent, Y-shaped oral opening (Fig. 1, Fig. 4A). Amphids lateral, four cephalic papillae sub-median (Fig. 4D). Esophagus with a neodont bearing two pairs of denticles lateroventral (Fig. 1B and C). Nerve ring, excretory pore, and deirids papilla-like-shaped in the anterior region between the nerve ring and excretory pore (Fig. 4B and C).

**Fig. 2:**
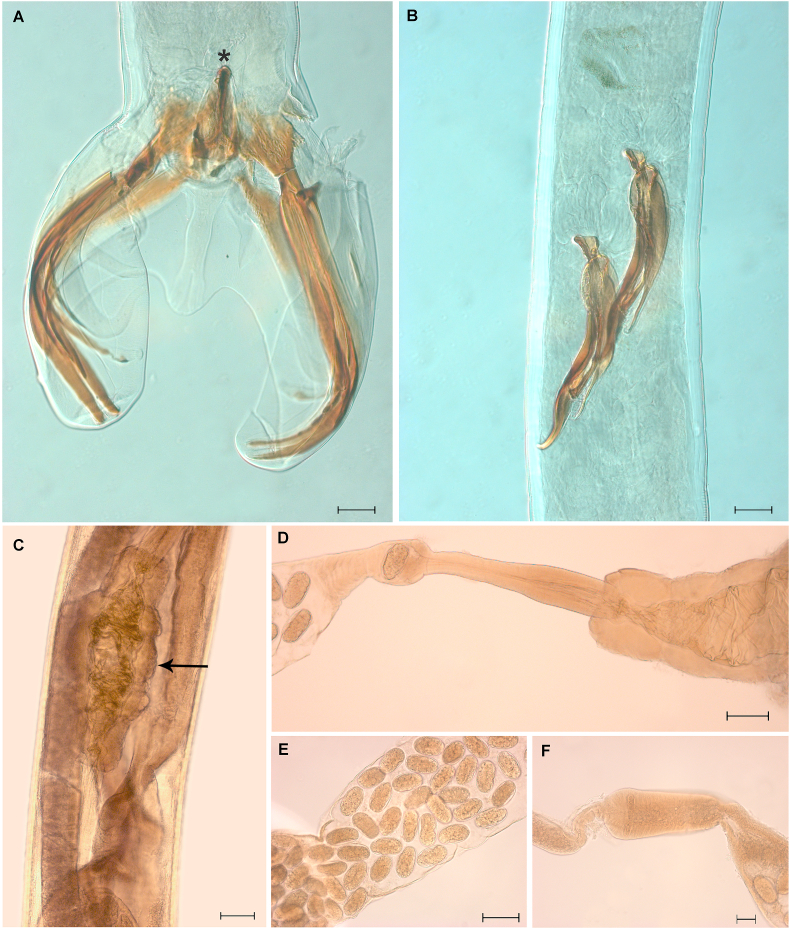
Light microscopy of *Boehmiella wilsoni*. (A) Posterior part of male body, gubernaculum (asterisk). (B) Spicule. (C) Posterior part of female body, vulva (arrow). (D) Dissected ovejector. (E) Uterus with eggs. (F) Spermatheca.

**Fig. 3:**
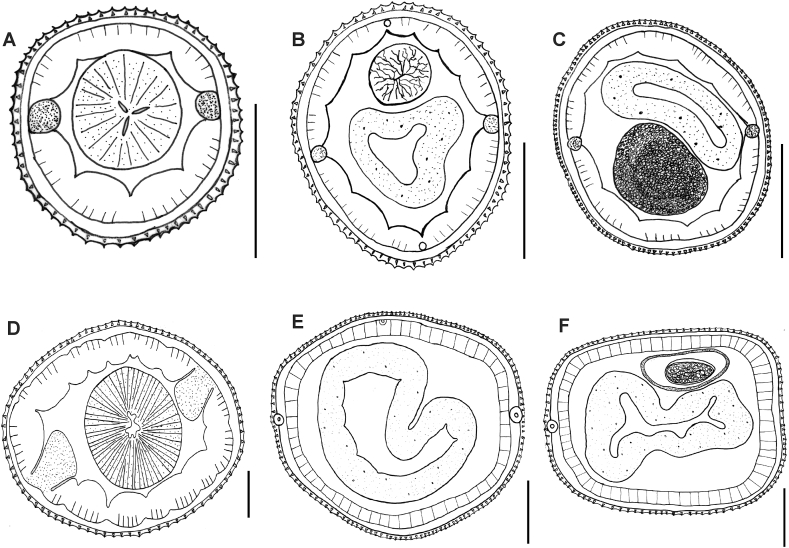
Light microscopy with camera lucida of *Boehmiella wilsoni*. (A) Cross-section of the body in the cervical region. (B) Cross-section of the body in the middle region. (C) Cross-section of the body in the posterior extremity (Female). (D) Cross-section of the body in the cervical region. (E) Cross-section of the body in the middle region. (F) Cross-section of the body in the posterior extremity (Male).

**Fig. 4:**
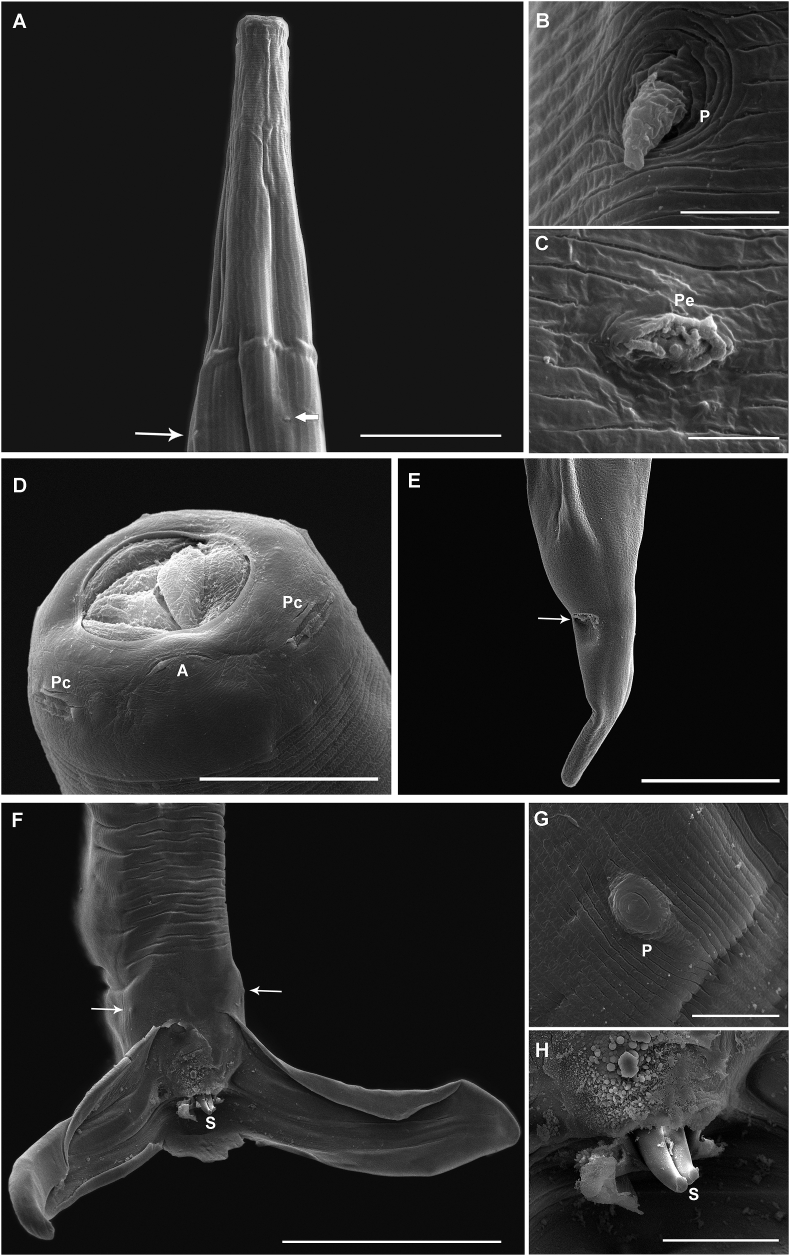
Scanning electron microscopy of *Boehmiella wilsoni*. (A) Anterior region showing a deirid (arrowhead) and the excretory pore (arrow). (B) Detail of a deirid. (C) Detail of excretory pore. (D) Anterior end in apical view showing two cephalic papillae (pc) and amphid (a). (E) Posterior end of female and detail of anus (arrow). (F) Posterior end of male, showing the prebursal papillae (arrow) and spicule tip(s). (G) Detail of a prebursal papillae (p). (H) Detail of a pair of spicule tips. Abbreviation: v-ventral and d-dorsal.

Males with well-developed pair of prebursal papillae (Fig. 4F and G) and an asymmetrical trilobate bursa with reduced dorsal lobe (Fig. 1, Fig. 2, Fig. 4F). Internal dorsal ray (9) bifurcated distally, and external ray (10) bidigitate tips (Fig. 1F). The posterolateral (4), mediolateral (5) and externolateral (6) rays highly sclerotised. A gubernaculum present, and telamon slight sclerotization, organized in three interconnected parts (Fig. 1H and I). Spicule short, complex, and sclerotised, with three tips (Fig. 2, Fig. 4H).

Females with didelphic uterus, ovejector with two branches and a vulva in the posterior third of the body (Fig. 1, Fig. 2C, D), with numerous thin-shelled hyaline larvae eggs containing (Fig. 2E). The anus near end of the body, with a sharp tail, but without spine (Fig. 4E). Spermatheca present with spermatozoa (Fig. 2F).

Female synlophe bearing 81 ridges at the cervical region, with 142 ridges in the mid-region, and 90 in the posterior extremity. Male synlophe bearing 64 ridges at the cervical region, with 126 ridges in the mid-region, and 78 in the posterior extremity (Fig. 3A–F).

Measurements recorded in this study were compared with Gebauer (1932), Lucker (1943), and Mollericona (2016) descriptions (Table 2) and indicate that the specimens were *B. wilsoni*.

**Table 2:** Measurements in millimeters of male and female specimens of genus *Boehmiella* from original descriptions found in the literature and from the present study.

	*B. perichitinea* Gebauer (1932)	*B. wilsoni* Lucker (1943)	*B. wilsoni* Mollericona et al. (2016)	*B. wilsoni* Present study
Locality	Germany	United States	Bolivia	Acre, Brazil
Host	*Myocastor coypus*	*Sciurus carolinensis*	*Dasyprocta variegata*	*Mesomys hispidus*
**Male**				**(n** = **10)**
Length (L)	15–18	17.1–20.3	13.6–17,3	15.8–19.5
Width (W)	0.24	0.21–0.24	0.21–0.29	0.18–0.21
Esophagus	–	0.84–0.94	0.86–1.01	0.87–0.96
Nerve-ring	–	0.32–0.45	0.28–0.31	0.29–0.48
Excretory pore	–	–	0.32–0.39	0.37–0.57
Bursal Types	2-1-2	2-1-2	2-1-2	2-1-2
Spicule	0.26	0.30–0.32	–	0.29–0.31
Gubernaculum	0.1	0.12–0.14	–	0.11–0.14
Telamon	Absent	Present	Present	Present
Cloaca (L)	–	–	–	0.22–0.24
Dorsal rays (L)	0.2	–	–	0.17–0.18
Lateral rays (L)	Sclerotised	Sclerotised	Sclerotised	Sclerotised
**Female**				**(n** = **10)**
Length (L)	21–25	37.3–43.3	32.4–39.7	39.3–45.9
Width (W)	0.24–0.31	0.40–0.58	0.37–0.50	0.34–0.46
Esophagus	–	1.00–1.29	1.22–1.49	1.17–1.39
Nerve-ring	–	–	0.34–0.42	0.34–0.36
Excretory pore	–	–	0.35–0.45	0.48–0.49
Vulva	–	–	–	7.88–9
Anus	–	–	–	0.38–0.46
Tail	0.32–0.37	–	–	0.38–0.46
Anterior region				
Vagina vera	–	–	–	0.09–0.14
Vestibule	–	–	–	0.09–0.14
Sphincter (L x W)	–	–	–	0.11 × 0.06
Infundibulum	–	–	–	0.40–0.66
Uterus	–	–	–	4.1–4.9
Posterior region				
Vagina vera	–	–	–	0.07–0.14
Vestibule	–	–	–	0.10–0.12
Sphincter (L x W)	–	–	–	0.10 × 0.06
Infundibulum	–	–	–	0.46–0.70
Uterus	–	–	–	4.3–6.9
Eggs (L x W) (μm)	–	88–105 × 50-62	87.5–92.5 × 52.5	85–98.4 × 49.5–54

#### 3.1.1 Taxonomic summary

*Boehmiella wilsoni* Luker, 1943.

Host: *Mesomys hispidus* Desmarest, 1817.

Site of infection: stomach.

Location: Fazenda Experimental Catuaba, municipality of Senador Guiomard, state of Acre, Brazil (10°09′39.0″ S; 67°44′17.6″ W).

Prevalence: 33% (1 rodent positive in 3 rodents examined).

Intensity of infection: 29 (29 helminth specimens/1 positive rodent).

Abundance: 9.7 (29 helminth specimens/3 rodents collected).

Specimens: 2 voucher (1 male and 1 female) deposited in the Helminthological Collection of Oswaldo Cruz Institute, Rio de Janeiro, Brazil (*Coleção Helmintológica do Instituto Oswaldo Cruz*) under N° CHIOC38568.

#### 3.1.2 Genus B*oehmiella*

Diagnosis: Boehmiellidae: buccal capsule and cephalic vesicle absent, well-developed neodont emerging from the anterior part of the esophagus, two pairs of denticles in its lumen; lateral rays highly sclerotised; gubernaculum present; vulva posterior.

#### 3.1.3 Family Boehmiellidae fam. nov

Diagnosis: Heligmosomoidea: buccal capsule absent, neodont followed by two pairs of minute denticles; copulatory bursa asymmetrical trilobed, reduced dorsal lobe; lateral rays highly sclerotised, spicules short and complex; gubernaculum present. Female tail without spine, vulva posterior; didelphic; single genus: *Boehmiella* Gebauer (1932).

### 3.2 Molecular analyses

The amplification of partial 18S rRNA gene of *B. wilsoni* yielded two sequences with good quality chromatograms, which we assembled into a contig of 794 base pairs (bp). The amplification of partial 28S rRNA gene of *B. wilsoni* yielded 24 sequences with good quality chromatograms, which we assembled into a contig of 2,734 bp. The amplification of partial 28S rRNA gene of *Viannaia hamata* yielded four sequences with high-quality chromatograms, which we assembled into a contig of 1,285 bp. The 18S rRNA gene sequences from the present study, aligned with those retrieved from GenBank, resulted in a matrix of 27 taxa and 794 characters (Supplementary File 1). From these, 717 characters were constant and 50 were parsimony informative. The 28S rRNA gene sequences from this study, aligned with those retrieved from GenBank, resulted in a matrix of 37 taxa and 1,293 characters (Supplementary File 2). From these, 1,218 characters were constant and 36 were parsimony informative. Both matrices had strong phylogenetic signals conveyed by PTP and G1 tests (Supplementary File 3 and 4) and little evidence of nucleotide substitution saturation conveyed the test by Xia et al. (Supplementary File 5 and 6). The matrix of concatenated 28S and 18S rRNA sequences included 16 taxa (Table 1) and 3874 characters. Overall, 3,527 of these characters were constant and 171 were parsimony informative.

For all matrices, the PhyML-SMS selected the GTR+G +I as the best-fit nucleotide substitution model for the data, with optimized ML frequencies, and four rate categories. In the 18S matrix, we used an estimated Gamma-shape parameter of α = 0.116 and a proportion of invariable sites of 0.431. The 18S best log-likelihood ML-tree score was −2007.293666. In the 28S matrix, we used an estimated Gamma-shape parameter of α = 0.719 and a proportion of invariable sites of 0.883. The 28S best log-likelihood ML-tree score was −2658.831348. For the concatenated matrix, we used an estimated Gamma-shape parameter of α = 0.591 and a proportion of invariable sites of 0.819. The concatenated best log-likelihood ML-tree score was −8936.767284.

In the BI, substitution models selected by PAUP × were the TVM+I+G, for the 18S matrix (Supplementary File 7), and the K80+I+G, for the 28S matrix (Supplementary File 8). For the concatenated matrix, we used the HKY+I and the TVM+I+G models for the 18S and 28S partitions, respectively, with unlinked parameters (Supplementary File 9). For the 18S matrix, the BI mean estimated marginal likelihood was −2018.2782 and the median was −2017.956. The 18S ESSs were above 121 for all parameters. For the 28S matrix, the BI mean estimated marginal likelihood was −2717.1052 and the median was −2716.765. The 28S ESSs were above 106 for all parameters. For the concatenated matrix, the BI mean estimated marginal likelihood was −8955.9273 and the median was −8955.601. The concatenated ESSs were above 21.624 for all parameters.

The pairwise uncorrected *p*-distances calculated for each matrix are summarized in the Supplementary Files 10 and 11. Across the 18S gene matrix, pairwise *p*-distances ranged from 0.1%, between *Nicollina cameroni* Thomas, 1959 and *Austrostongylus victoriensis* Cassone, 1983, to 4.4%, between *Vexillata convolute* Caballero and Cerecero, 1943 and *Necator americanus*. *B. wilsoni p*-distances, against the other 18S gene sequences, ranged from 1.1% (*A. victoriensis*) to 3.9% (*Nematodirus battus* Crofton and Thomas, 1951). Pairwise 18S *p*-distances between *B. wilsoni* and the Haemonchidae ranged from 3.3% (*Haemonchus contortus* Rudolphi, 1803) to 3.6% (*Ostertagia ostertagi* Stiles, 1892). Within the family Haemonchidae, 18S genetic distances ranged from 0.5%, between *Ostertagia leptospicularis* Asadov, 1953 and *O. ostertagi*, to 1.7%, between *H. contortus* and *O. ostertagi*, with a mean *p*-distance of 1.3%. Within Viannaiidae Durette-Desset and Chabaud, 1981, 18S genetic distances ranged from zero, between *Viannaia minispicula* Guerrero, 1985 and *V. hamata*, and between *Travassostrongylus orloffi* Travassos, 1935 and *T. callis* Travassos, 1914, to 2.4%, between *Oswaldocruzia* Travassos, 1917 and *Viannaia didelphis* Travassos, 1914 (mean = 1.3%).

Across the 28S gene matrix, pairwise *p*-distances ranged from zero, between *Teladorsagia circumcincta* (Stadelmann, 1894) with *Hyostrongylus rubidus* (Hassall and Stiles, 1892), *Patricialina hickmani* (Mawson, 1973) with *Paraustrostrongylus bettongia* Mawson, 1973, *Nematodirus helvetianus* May 1920 with *N. battus*, and *Odilia bainae* Beverige and Durette-Desset, 1992 with *Nippostrongylus magnus* Mawson, 1961, to 2.1%, between *Ollulanus tricuspis* Leuckart, 1865 and *Nippostrongylus brasiliensis* Travassos, 1914. *B. wilsoni p*-distances, against the other 28S gene sequences, ranged from 0.4% (*N. cameroni*) to 1.5% (*O. tricuspis*). Pairwise 28S *p*-distances between *B. wilsoni* and the Haemonchidae ranged from 0.7% (*Teladorsagia circumcincta* and *H. rubidus*) to 1.2% (*H. contortus*). Within the family Haemonchidae, 28S genetic distances ranged from 0.0%, between *T. circumcincta* and *H. rubidus*, to 1.3%, between *H. contortus* and *O. leptospicularis* (mean = 0.6%). In the family Hepertostrongylidae (Skrjabin and Schulz, 1937), interspecific distances in the 28S matrix ranged from zero, between *P. hickmani* and *P. bettongia*, to 0.8% between *Hepertostrongylus python* Baylis, 1931, with both *Amphicephaloides thylogale* Beveridge, 1979 and *Globocephaloides macropodis* Yorke and Maplestone, 1926.

**Fig. 5:**
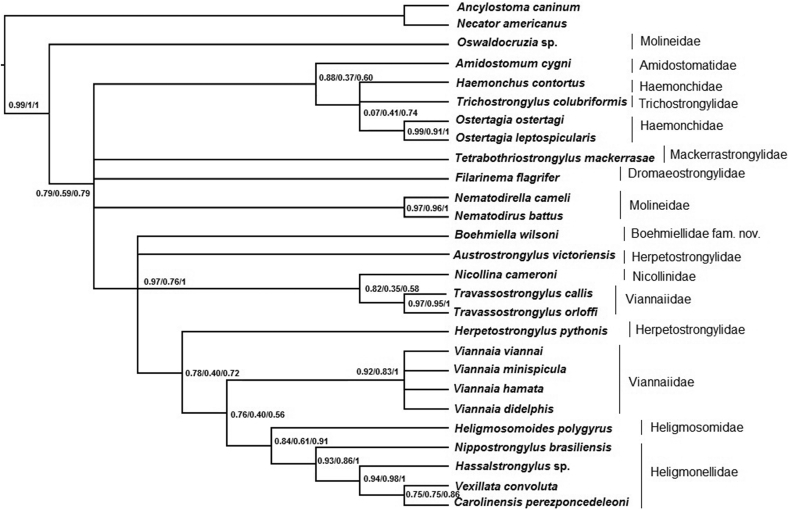
18S gene matrix strict consensus cladogram of ML and BI analyses.

**Fig. 6:**
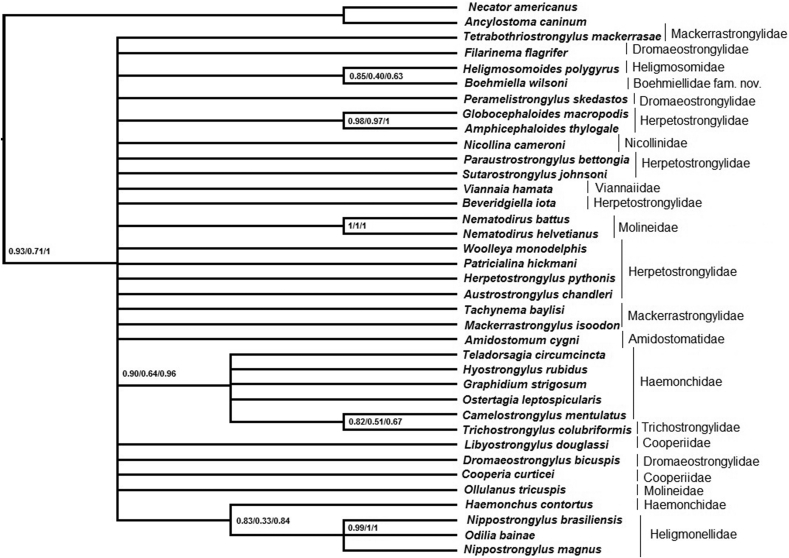
28S gene matrix strict consensus cladogram of ML and BI analyses.

**Fig. 7:**
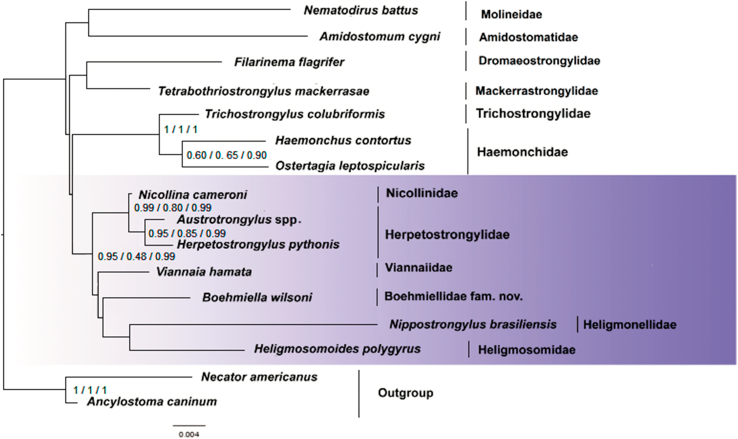
Phylogenetic relationships of *Boehmiella wilsoni*, Trichostrongylina, and outgroup sequences. Concatenated 18S and 28S genes matrix ML phylogram. Support values at nodes: aLRT/ML-BP/BPP, respectively.

The ML and BI phylogenies had similar topologies, with little variation in the nodes or support values, for each matrix (Supplementary Files 12–20). All phylogenetic reconstructions recovered Trichostrongylina as monophyletic with high support values. For the 18S and 28S gene matrices, the ML and BI phylogenies were summarized in a strict consensus tree for each matrix (Fig. 5, Fig. 6). The concatenated 18S and 28S genes matrix ML-phylogenetic tree is shown in Fig. 7, which summarizes node supports found in the ML and BI phylogenetic analyses. Both topologies showed the concatenated 18S and 28S genes sequence of *Boehmiella* nesting within a poorly-to-strongly supported (aLRT = 0.95, ML-BP = 0.48, BPP = 0.99) monophyletic group with representative sequences of the families Heligmonellidae (Skrjabin and Schikhobalova, 1952), Heligmosomidae (Travassos, 1914), Herpetostrongylidae, Nicollinidae (Skrjabin and Schulz, 1937), and Viannaiidae Neveu-Lemaire, 1944. We will refer to this monophyletic group as Clade 1. Although most relationships within Clade 1 were poorly supported, Nicollinidae was sister, with strong support (aLRT = 0.99, ML-BP = 0.80, BPP = 0.99), to a well-supported monophyletic family Herpetostrongylidae (aLRT = 0.95, ML-BP = 0.85, BPP = 0.99), forming an Australasian clade. *Boehmiella* was sister, with little support (aLRT = 0.15, ML-BP = 0.17, BPP = 0.57), to a poorly-to-moderately supported clade formed by sequences of Heligmonellidae and Heligmosomidae representatives (aLRT = 0.83, ML-BP = 0.30, BPP = 0.55). That Heligmonellidae-Heligmosomidae-*Boehmiella* clade was sister to Viannaiidae, also with support values ranging from little to moderate (aLRT = 0.83, ML-BP = 0.30, BPP = 0.55).

Three other clades were recovered in our analyses, although their supports varied largely. Clade 2, formed by a moderately-supported monophyletic Haemonchidae (aLRT = 0.60, ML-BP = 0.65, BPP = 0.90), sister to Trichostrongylidae with strong support (aLRT = 1.00, ML-BP = 1.00, BPP = 0.99); Clade 3, formed by representatives of the families Dromaeostrongylidae Durette-Desset, 1983 and Mackerrastrongylidae (Inglis, 1968), although with little-to-moderate support (aLRT = 0.83, ML-BP = 0.20, BPP < 0.50); and Clade 4, formed by representatives of the families Amidostomatidae (Travassos, 1919) and Molineidae (Skrjabin and Schulz, 1937), also with little-to-moderate support (aLRT = 0.75, ML-BP = 0.29, BPP = 0.57). The relationships among the four trichostrongylin clades, recovered in our analyses, were poorly resolved and Trichostrongylina would be better represented as a polytomy.

## 4 Discussion

The morphological characters that differentiate *B. wilsoni* from its congener *B. perichitinea* include the size prebursal papillae, the presence of a telamon, the absence of a cervical wing (structure observed in the lateral body), a larger gubernaculum, larger female, and the larger number of ridges in the synlophe (Luker, 1943). While the morphology and morphometry of *Boehmiella* have been studied previously, the reproductive tract of the female has not been described adequately, until now. In this study, we were able to provide the first measurements of several internal structures of the reproductive apparatus of the females.

Durette-Desset and Sutton (1979) described the synlophe of *B. perichitinea* as having longitudinal ridges (28 in the male and 34 in the female) in the middle of the body. Particularly, they observed a set of three ridges in the anterior half of the body, which were spaced well apart from the other ridges. The center-most of these three ridges extends gradually along the posterior region of the body, while the two lateral ridges form wings toward the anterior region of the body.

The *B. wilsoni* specimens analyzed in this study had a large number of ridges, which contrasts with the observations of Falcon-Ordáz and García-Prieto (2004), who were the first to describe a synlophe in this species in the form of small ridges perpendicular to the longitudinal axis of the body. These authors observed 34 ridges at the level of cervical papillae in the female, 47 in the region where the intestine starts, 46 in the pre-equatorial portion of the body, and 22 in the mid-region. In the males, however, the ridges are limited to the mid-region of the body, although the authors do not provide information on their number.

The fact that *B. perichitinea* has a reduced number of ridges and a well-developed lateral ridge in its anterior region suggests that Falcon-Ordáz and García-Prieto (2004) may have not actually analyzed specimens of *B. wilsoni*. This is reinforced by the fact that their specimens had well-developed lateral ridges, a characteristic absent in *B. wilsoni*. These authors also identified their specimens using characters that are diagnostic of the genus, i.e., the sclerotised lateral rays, spicules divided into branches, and the presence of a neodont, which are insufficient to determine the species. In the present study, the use of SEM and the analysis of the cross-sections of the body allowed us to verify the details of the number and pattern of the ridges in the synlophe of *B. wilsoni*, specially, to confirm that both male and female specimens have a larger number of ridges than that reported by Falcon-Ordáz and García-Prieto (2004).

Durette-Desset et al. (1999) provide an in-depth cladistic analysis of the superfamily Trichostrongyloidea Cram, 1927, in which *Boehmiella* is allocated to the family Haemonchidae (subfamily Haemonchinae), even though some characteristics of this genus are distinct from those of the haemonchids. The diagnostic morphological traits of the haemonchids are the ungrouped rays of the lateral trident, the presence of externolateral ray (4) and/or externodorsal ray (8) reaching the edge of the caudal bursa, and hook-shaped deirids. The characteristics of the Haemonchinae are the type 2-1-2 caudal bursa, the mediolateral (5) and posterolateral (6) rays that are either joined or parallel, the ventro-ventral (2) and lateroventral (3) rays with a long common trunk, and the hexagonal buccal opening connected laterally to a hexagonal ring (Durette-Desset et al., 1999; Durette-Desset and Digiani, 2012).

By contrast, *Boehmiella* has a triangular, Y-shaped oral opening, with one neodont and four denticules the anterior portion of the esophagus, which is an autapomorphic characteristic for the genus, given that the oral opening of the haemonchids is hexagonal and has only a single neodont. The hook-shaped deirids are a synapomorphic characteristic to the haemonchid genera, but under scanning electron microscopy, the deirids observed in *Boehmiella* were modified and papilla-like in shape and did not show the characteristic hook-shape of the haemonchids.

The type 2-1-2 caudal bursa (Durette-Desset and Digiani, 2012), the ungrouped rays of the lateral trident, and the joined and parallel rays mediolateral (5) and posterolateral (6) are characters common to *Boehmiella* and the Haemonchidae. Externolateral ray (4) and externodorsal ray (8) extending to the edge of the caudal bursa is an apomorphic characteristic of the Strongylida (Durette-Desset et al., 1999). However, in *Boehmiella* externolateral ray (4) and externodorsal ray (8) are short, which suggests a plesiomorphic character of the Strongylida, also found in the Trichostrongylinae. *Boehmiella* has smaller than lateroventral (3) rays, like the Ostertagiinae but shows the separation of the rays at half length. In *Boehmiella,* the distal ends of ventro-ventral (2) and lateroventral (3) rays are curved and pincer-like with greater distance between the extremities, and like the Cooperiidae, which differentiate *Boehmiella* from the haemonchids. The sclerotization of the lateral rays is a characteristic exclusive to *Boehmiella*.

Gibbons and Khalil (1982) differentiated the genus *Boehmiella* from other haemonchid genera by the presence of more than one tooth in the buccal cavity and the esclerotization of the lateral rays of the caudal bursa. Moreover, the presence of a neodont in the oral cavity appears to be homoplastic, given that the genera of other trichostrongyloid families (e.g., the Mackerrastrongylidae) also show this characteristic. The esclerotization of the lateral rays is exclusive to the genus *Boehmiella* in the superfamily Heligmosomoidea and therefore, it may represent an autapomorphy.

Our molecular analyses suggested that the genus *Boehmiella* does not belong to the family Haemonchidae, as proposed by Durette-Desset et al. (1999), neither the Trichostrongylidae (*sensu* Durette-Desset, 1985). Rather, *Boehmiella* was more closely related to other families, such as Heligmonellidae, Heligmosomidae, Viannaiidae, Nicollinidae and the Herpetostrongylidae, forming a polytomous clade (Clade 1) in different topologies in different analyses. Moreover, the results of our molecular phylogenies indicated that the genus *Boehmiella* belongs to a family distinct from the Australasian trichostrongylins of the families Herpetostrongylidae that are known from Australian marsupials and reptiles in Australia and south-east Asia, and Nicollinidae which occurs in monotremes. Chilton et al. (2015) demonstrated for the first time the close phylogenetic relatedness between Herpetostrongylinae and Nicollinidae, a result also found in our analyses. However, *Boehmiella* does have a number of morphological traits that are found in herpetostrongylids and nicollinids, such as the robust esophageal neodont, and the complex spicules and reduced dorsal lobe in the caudal bursa, which we interpret as simplesiomorphic characters shared by the Boehmiellidae fam. nov., Herpetostrongylidae, and Nicollinidae. As for the other families that formed a polytomous clade with *Boehmiella*, this genus shares an absence of spine in the female tail with the Heligmonellidae and the 2-1-2 type of copulatory bursa with some viannaiid genera. However, no morphological feature is shared with the family Heligmosomidae.

Reconstructing the origin and diversification of the Superfamily Heligmosomoidea is a challenging task. Some families are widely distributed (Heligmonellidae, Heligmosomidae, Ornithostrongylidae), while others are more resctricted (Herpetostrongylidae, Nicollinidae, Viannaiidae) (Durette-Desset, 2009; Beveridge et al., 2014; Durette-Desset, 1985). Most families have Neotropical genera (Heligmonellidae, Nicollinidae, Ornithostrongylidae), fewer have Australasian (Heligmonelidae, Nicollinidae, Herpetostrongylidae) or Holarctic (Heligmonellidae, Heligmosomidae, Ornithostrongylidae) genera (Durette-Desset, 2009; Beveridge et al., 2014; Durette-Desset, 1985). As for the hosts, most families have rodents for hosts (Heligmonellidae, Heligmosomidae, Ornithostrongylidae, Viannaiidae), caviomorph rodents are hosts for two of them (Heligmonellidae, Viannaiidae), and marsupials are hosts for three families (Herpetostrongylidae, Nicollinidae, Viannaiidae) (Durette-Desset, 2009; Beveridge et al., 2014; Durette-Desset, 1985). The Clade 1, formed by Boehmiellidae fam. nov., Heligmonellidae, Heligmosomidae, Herpetostrongylidae, Nicollinidae, and Viannaiidae, supports the inclusion of Boehmiellidae fam. nov. within the Superfamily Heligmosomoidea.

Beveridge and Spratt (2015) suggest a Gondwanan component associated to the possible connection between families Viannaiidae in South American marsupials and Herpetostrongylidae in Australasian marsupials observed by Beveridge and Spratt (1996) and Humphery-Smith (1983), as well as other parasites, such as cestodes occurring on both continents (Beveridge and Spratt, 2015). Durette-Desset (1985) points to similarities of the synlophe in the Herpetostrongylidae and Viannaiidae, consisting of three ventral left ridges (characteristic also shared with Heligmosomidae) and the oblique axis of orientation, although these may be plesiomophically-shared conditions. Durette-Desset (1985) also points that the primitive Viannaiidae infected Neotropical marsupials, probably arising during the Eocene, later spreading to caviomorph rodents in the upper Eocene. This was corroborated by the close relationship between Australasian and Neotropical trichostrongylins that we found. In fact, evidences suggest that intense and dynamic processes of migrations, dispersal, radiations, and vicariance of vertebrates took place between South America and Australia, through Antarctica in both directions during the Gondwanan break-up 160–30 Ma (Beck et al., 2008; Nilsson et al., 2010; Upchurch, 2008). Some heligmosomoid lineages may have differentiated before the separation of South America and East Gondwana ~80 Ma (Upchurch, 2008). Nevertheless, since it is conceivable that the marsupial colonization of South America from North America took place between 75 and 65 Ma (Nilsson et al., 2004; Williamson et al., 2014), we may presume that latter heligmosomoid families emerged sometime between 50 and 35 Ma, preceding dispersion across narrow seaways, prior to the final break-up (Upchurch, 2008).

Durette-Desset and Sutton (1979) suggest that the genus *Boehmiella* is the first evolutionary line of the Haemonchidae, and subsequently Durette-Desset et al. (1999) postulate that during the Upper Miocene the differentiation of *Boehmiella* coincided with the entry of squirrels in North America, later parasitizing the caviomorph *Myocastor*. We argue that the reverse may have occurred: It is more likely that the lineage leading to *Boehmiella* differentiated by infecting Neotropical caviomorph rodents, as well as some viannaiids and heligmonellids, during the Upper Eocene or Lower Oligocene; infected Nearctic sciuromorph rodents after the Great American Biotic Interchange (GABI); and only very recently reached the Holarctic region with invading *M. coypus*.

To address all those hypotheses, future studies on the evolution of Heligmosomoidea would need to rely on larger databases and benefit from a framework based on molecular clock approaches, as the one used for Ascaridoidea by Li et al. (2018).

Overall, although only a limited number of trichostrongylin taxa were available, for both genes, in this study, some findings are conclusive. The genus *Boehmiella* is clearly unrelated to the family Haemonchidae. Given this, we propose a new family, Boehmiellidae fam. nov., which includes a single genus, *Boehmiella*, based on its morphological and molecular distinctiveness.

## Declaration of competing interest

The authors declare that they have no known competing financial interests or personal relationships that could have appeared to influence the work reported in this paper.
